# Modeling Longitudinal Preclinical Tumor Size Data to Identify Transient Dynamics in Tumor Response to Antiangiogenic Drugs

**DOI:** 10.1002/psp4.12142

**Published:** 2016-11-14

**Authors:** LG Hutchinson, H‐J Mueller, EA Gaffney, PK Maini, J Wagg, A Phipps, C Boetsch, HM Byrne, B Ribba

**Affiliations:** ^1^ Wolfson Centre for Mathematical Biology, Mathematical Institute, University of OxfordOxfordUK; ^2^ Pharma Research and Early Development, Roche Innovation Centre MunichMunichGermany; ^3^ Roche Pharmaceutical Research & Early Development, Roche Innovation CenterBaselSwitzerland; ^4^ Pharma Research and Early Development, Roche InnovationWelwyn Garden CityUK

## Abstract

Experimental evidence suggests that antiangiogenic therapy gives rise to a transient window of vessel normalization, within which the efficacy of radiotherapy and chemotherapy may be enhanced. Preclinical experiments that measure components of vessel normalization are invasive and expensive. We have developed a mathematical model of vascular tumor growth from preclinical time‐course data in a breast cancer xenograft model. We used a mixed‐effects approach for model parameterization, leveraging tumor size data to identify a period of enhanced tumor growth that could potentially correspond to the transient window of vessel normalization. We estimated the characteristics of the window for mice treated with an anti‐VEGF antibody (bevacizumab) or with a bispecific anti‐VEGF/anti‐angiopoietin‐2 antibody (vanucizumab). We show how the mathematical model could theoretically be used to predict how to coordinate antiangiogenic therapy with radiotherapy or chemotherapy to maximize therapeutic effect, reducing the need for preclinical experiments that directly measure vessel normalization parameters.

**Study Highlights**

**WHAT IS THE CURRENT KNOWLEDGE ON THE TOPIC?**

☑ Antiangiogenic therapies alter the density and architecture of the tumor blood vessel network, and may stimulate a transient window of vessel normalization shortly after antiangiogenic treatment commences. The efficacy of chemotherapy and radiotherapy may be enhanced during the transient window due to increased perfusion and decreased vascular permeability.

**WHAT QUESTION DOES THIS STUDY ADDRESS?**

☑ Can tumor size data alone be used to infer the transient window of vessel normalization, in which the efficacy of chemotherapy and radiotherapy may be enhanced?

**WHAT THIS STUDY ADDS TO OUR KNOWLEDGE**

☑ A transient window of enhanced tumor growth occurs during treatment with bevacizumab or vanucizumab for KPL‐4 tumor bearing mice. The window is identified with precision using mixed‐effects techniques.

**HOW MIGHT THIS CHANGE DRUG DISCOVERY, DEVELOPMENT, AND/OR THERAPEUTICS?**

☑ Identification of the transient window of enhanced tumor growth could reduce the need to measure normalization parameters, and could reduce the chance of obtaining inconsistent efficacy measurements when comparing treatments to be administered alongside antiangiogenic treatment.

Angiogenesis is the process by which blood vessels form from existing ones; it plays a key role in tumor growth and progression. The initial development of antiangiogenic therapies was based on the premise that pruning new tumor vessels would reduce the blood supply to the tumor, and inhibit the delivery of oxygen and nutrients to the tumor, causing its growth to slow down or stop.^1^ However, it is now clear that antiangiogenic therapy not only causes vascular regression, but also affects processes including vessel permeability, perfusion, diameter, tortuosity, and pericyte coverage; and thereby normalizes the vasculature.^2^ It has been suggested that vessel normalization plays a key role in tumor progression, since it may transiently enhance the delivery of oxygen and nutrients to the tumor microenvironment.^3^ There is evidence that antiangiogenic drug induced vessel normalization transiently increases the efficacy of chemotherapy and radiotherapy.^4^, ^5^ If this normalization window were identified for individual patients, then combination treatment schedules could be designed in which administration of chemotherapy or radiotherapy would be coordinated with the normalization window to maximize the therapeutic response.

Normalization has been observed in preclinical models of antiangiogenic therapy both from histology^6^, ^7^ and via real‐time imaging methods such as window chamber assays.^8^, ^9^ For reviews on the role of normalization in neovascular development, see refs. 10 and 11. Evidence from mouse xenograft studies suggests that vessel normalization is a transient effect that begins shortly after the onset of antiangiogenic therapy and ends a few days later.^12^, ^13^ Furthermore, some clinical studies are consistent with antiangiogenic therapy stimulating a reduction in vessel permeability for glioblastoma^14^ and rectal cancer.^15^ In both refs. 12 and 13, radiotherapy was found to be most efficacious when administered within the transient window of increased tumor oxygenation. In particular, in ref. 12 a synergistic tumor growth delay was observed when radiotherapy was administered 4–6 days after the first dose of antiangiogenic therapy. It has also been suggested that normalized vessels allow efficient delivery of chemotherapy since improved perfusion allows effective extravasation of small molecules.^5^, ^16^

Bevacizumab is an antivascular endothelial growth factor (VEGF) antibody that has been approved for treatment of numerous cancers including renal cell carcinoma, non‐small‐cell lung cancer, and colorectal cancer. Vanucizumab is a bispecific antibody that recognizes VEGF with one arm (based on bevacizumab) and angiopoietin‐2 (Ang‐2) with the other arm (based on LC06, an Ang‐2‐specific antibody). Vanucizumab is currently in phase II trials to treat locally advanced or metastatic solid tumors (NCT01688206), and in combination with FOLFOX for metastatic colorectal cancer (NCT02141295). It is thought that since both VEGF and Ang‐2 promote angiogenesis, simultaneous inhibition of the ligands will result in an additive or even synergistic effect on tumor vessel regression.^7^ Antiangiogenic therapies are principally used in combination with cytotoxic therapies or radiotherapy. It has been shown that the timing of such concomitant treatments is important for the therapeutic outcome in preclinical experiments.^4^, ^5^ In order to further elucidate some of the mechanisms by which antiangiogenic therapy can improve treatment outcome when administered alone or in combination with chemotherapy, we have developed a mathematical model of vascular tumor growth. Our model can be used to identify a period of enhanced tumor growth that could correspond to the vessel normalization window within which cytotoxic or radiotherapeutic efficacy may be increased.

The development of mathematical models of angiogenesis has been driven by experimental results. Continuous, discrete, and hybrid models can be used to represent vessel growth dynamics, and tumor growth, in one, two, or three dimensions and may account for intricate biological details^17^, ^18^, ^19^, ^20^, ^21^ (for a review, see ref. 22). Without suitable experimental data, these models can yield qualitative mechanistic insight; with appropriate data, the models can be validated and parameterized and, thereby, their predictive power increased. Mathematical models can also be used to investigate the impact of mechanistic perturbations to angiogenesis and to formulate hypotheses about optimal therapy regimens.

For model development and parameterization, nonlinear mixed‐effects (NLME) modeling enables a data‐driven approach.^23^ A maximum likelihood approach is used to estimate population and individual parameters from experimental data. The method has been used widely to integrate tumor growth data with ordinary differential equation (ODE) models that characterize tumor growth kinetics in the presence and absence of cytotoxic treatments.^24^, ^25^, ^26^, ^27^, ^28^ Typically these models comprise a term that represents an empirical tumor growth law (e.g., logistic, Gompertzian, or exponential growth), and another term to represent tumor growth inhibition due to chemotherapy. For example, in ref. 24 a two‐phase tumor growth law (exponential followed by linear) is modified to account for a cytotoxic therapy which acts directly to kill the tumor cells. This model is extended in ref. 29 to account for antiangiogenic therapy, which is assumed to indirectly slow the tumor's growth rate; however, there is no variable for the tumor's vascular density.

Building on these models, Hahnfeldt *et al*. proposed a simple model of vascular tumor growth in which the tumor and the vasculature are treated separately. Vascular density is assumed to regulate the equilibrium size of the tumor while the tumor is assumed to promote angiogenesis.^30^ In ref. 31, Ouerdani *et al*. develop a model of vascular tumor growth in which a logistic tumor growth law is assumed, and the equilibrium tumor size is the vessel‐dependent carrying capacity. The authors use preclinical and clinical data to parameterize the model in the presence and absence of the tyrosine kinase inhibitor pazopanib which blocks several kinases to interfere both with angiogenesis and tumor cell growth. Similarly, in ref. 27, Wilson *et al*. present a logistic tumor growth model with a dynamic carrying capacity term that is parameterized for administration of antiangiogenic therapy alone and in combination with chemotherapy. The authors use their model to predict an optimal time for the administration of chemotherapy following administration of antiangiogenic therapy. They suggest that vascular normalization could play a key role in the identification of the optimal treatment schedule, although normalization is neglected in their model.

It is clear that vessel normalization plays a key role in vascular tumor growth, although parameters associated with normalization are rarely measured. While existing mixed‐effects models have not explicitly considered the dynamics of vessel normalization, in this article we are motivated by the transient dynamics apparent from the experimental data in the KPL‐4 preclinical mouse (xenograft) model of breast cancer to extend existing mathematical models of vascular tumor growth under antiangiogenic therapy to account for these dynamics. Our primary goal is to combine mixed‐effects modeling with tumor size data from KPL‐4 mouse xenografts to characterize the transient window of increased tumor growth following exposure to antiangiogenic therapy. The same model could be used to characterize normalization in clinical studies. To conclude our study, we demonstrate how the model can be used to predict the optimal time, relative to the transient window, to schedule chemotherapy.

## METHODS

### Preclinical data

Female SCID beige mice, age 8 weeks, were housed in specific‐pathogen‐free conditions according to committed guidelines (GV‐Solas, Felasa, TierschG) and injected with a suspension of 3 × 10^6^ KPL‐4 tumor cells into the right, penultimate, inguinal mammary fat pad. Treatment started 38 days after tumor cell injection, when tumors had reached a mean size of 70 mm^3^, and mice were randomized into control (omalizumab), bevacizumab treatment, and vanucizumab treatment groups with 10 mice per group.

Bevacizumab is an anti‐VEGF antibody, vanucizumab is a bispecific anti‐VEGF/anti‐Ang‐2 antibody that neutralizes both ligands, and omalizumab was included as an isotype control. Each antibody was administered via i.v. injection at a dose of 10 mg/kg, once per week, starting 38 days after inoculation for a total of 5 weeks.

**Figure 1:**
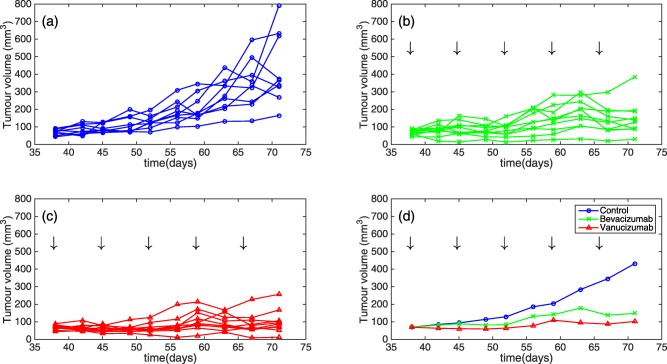
Individual tumor size data for (**a**) control tumors (*n* = 10), (**b**) bevacizumab treated animals (*n* = 10), (**c**) vanucizumab treated animals (*n* = 10), (**d**) mean tumor volume of individual groups. Treatment times are shown by vertical arrows in plots (**b–d**).

Tumor volume, *T*, was calculated using the formula $T=\frac{\text{length}\times {\text{width}}^{2}}{2}$, where the length and width of the tumor were the longest and shortest dimensions of the tumor lying at 90 ° to the longest, respectively. These measurements were taken twice per week for the treatment period, resulting in a total of 10 tumor size measurements per animal. The data are presented in **Figure** 1.

### Model development

#### Untreated tumors

The structural model that we present comprises two ordinary differential equations (ODEs) to describe time‐dependent tumor growth and associated vessel‐dependent carrying capacity and is inspired by a model devised by Hahnfeldt *et al*.^30^ The tumor size is the observed variable; the vessel‐dependent carrying capacity has not been measured experimentally. We assume that all individual parameters are distributed log‐normally, which is generally accepted for growth rates and reaction rates.^32^

The biological interpretation of the carrying capacity is the maximum tumor size that can be supported by the associated vasculature. The antiangiogenic therapies that we consider affect vessel growth, but are not directly cytotoxic. Therefore, we view tumor volume, *T* (measured in mm^3^), and the carrying capacity, *V* (also in mm^3^), as distinct dependent variables. A simple logistic growth model for *T* was chosen for the untreated case (see Eq. (1)). Tumor growth was represented using a logistic or generalized logistic growth term in several related models.^25^, ^27^, ^31^ Following ref. 30, we assume that the carrying capacity, *V*, of the tumor depends on the local vascular density and architecture. In recent publications,^27^, ^31^ the evolution of the carrying capacity was assumed to depend only on *T*. We propose that it is realistic to take into consideration the existing vascular density, since new vessels grow from existing ones. The untreated model of tumor size and dynamic carrying capacity can be written:

(1) $$\frac{dT}{dt}=\alpha_{T}\;T\left(1-\frac{T}{V}\right),$$

(2) $$\frac{dV}{dt}=\alpha_{V}\;T^{\beta }\;V^{\gamma },$$

where *α_T_* is the maximal tumor growth rate, and *α_V_* is the growth rate of the vascular‐dependent carrying capacity. Since in Eq. (2) the exponents *β* and *γ* are not identifiable via model simulations, we fixed them at physiologically based values. Vessel growth is stimulated by growth factors (such as VEGF) that are released by tumor cells in response to hypoxia and, by a simple geometrical argument, we may assume that the proportion of the tumor volume that is hypoxic is proportional to its surface area. This assumption is consistent with the pO2 gradient in tumors described previously.^33^ Accordingly, we fix $\beta =\frac{2}{3}$. We use a value of $\gamma =1-\beta =\frac{1}{3}$ to ensure that vessel growth is exponential at long times. We note that there are several values for *β* and *γ* that could feasibly represent vessel‐dependent tumor growth: the values chosen here are based on geometrical arguments.

#### Tumors treated with antiangiogenic therapy

We assume that antiangiogenic therapy has two effects: (1) it causes blood vessel regression and (2) it leads to a transient period during which tumor growth is enhanced due to increased blood flow. We start by taking only effect (1) into account, and then suppose both effects are active. We incorporate effects (1) and (2) into our mathematical model by including a term for vessel regression and a function that transiently increases the carrying capacity (this change being stimulated by increased tumor perfusion during the transient normalization window).

In the absence of pharmacokinetic data from the experiment and to avoid introducing further unknown model parameters, we do not explicitly model drug levels in the tumor microenvironment; we assume that from the onset of treatment, the concentration of each antiangiogenic drug in the blood stream is maintained at a high enough level to ensure maximum efficacy. The long half‐life (around 8 days in preclinical models) of bevacizumab supports this assumption,^34^ and we assume that vanucizumab has a similarly long half‐life. Data presented in refs. 34 and 7 show that a weekly dose of 10 mg/kg of bevacizumab or vanucizumab results in maximum efficacy with respect to tumor growth inhibition in mice, further supporting our argument. The full model can be written:

(3) $$\frac{dT}{dt}=\alpha_{T}\;T\left(1-\frac{T}{N\times V}\right),$$

(4) $$\frac{dV}{dt}=\alpha_{V}\;T^{2/3}\;V^{1/3}-\delta_{V}\;V,$$

(5) $$N(t)=\{\begin{array}{cc} 1 & \text{for}\;t\leq t_{norm1}\;\text{and}\;t\geq t_{norm2},\\ N_{\max} & \text{for}\;t_{norm1}<t<t_{norm2}, \end{array}$$

where the constant *δ_V_* corresponds to the vessel death rate, *t_norm_*_1_ and *t_norm_*_2_ are the start and end times of the window of enhanced tumor growth, respectively, and $N_{\text{max}}\geq 1$ is the maximum factor by which the carrying capacity is enhanced during the transient window. For the first case, where only effect (1) is accounted for, *N_max_* = 1 for all time.

**Figure 2:**
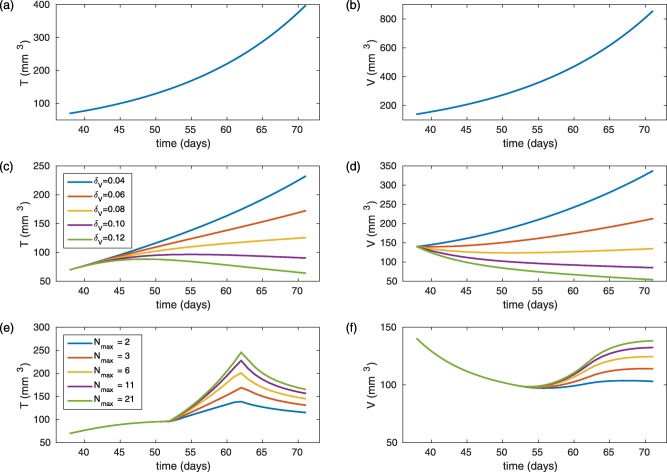
Typical model simulations with arbitrary parameters that are order of magnitude estimates. Curves on the left (**a,c,e**) represent the tumor volume *T*(*t*) and corresponding curves on the right (**b,d,f**) represent the vessel dependent carrying capacity *V*(*t*). (**a,b**) Control simulations (Eqs. 1, 2) with $\alpha_{T}=0.1{\;\text{day}}^{-1},\;\alpha_{V}=0.09{\;\text{day}}^{-1}$, *K* = 2, *T*_0_ = 70 mm^3^. (c,d) Treatment model simulations (Eqs. 3**–**5) with *N_max_* = 1, *δ_V_* values $0.04{\;\text{day}}^{-1}-0.12{\;\text{day}}^{-1}$ and all other parameters the same as (a,b). (e,f) Treatment model simulations (Eqs. 3**–**5) where *t_norm_*_1_ = 52 days, *t_norm_*_2_ = 62 days, $N_{\text{max}}\;\text{values}\;2-21,\;\delta_{V}=0.1{\;\text{day}}^{-1}$ and all other parameters the same as (a,b).

The initial condition for *T* is the experimental value of the tumor volume at the first measurement time (day 38). We estimate the initial value of *V* via the parameter $K=\frac{T_{0}}{V_{0}}$, where *T*_0_ is the observed initial tumor volume and *V*_0_ is the initial vessel‐dependent carrying capacity. As such, we assume that *V*_0_ is linearly related to *T*_0_ for individuals through the parameter *K*, which is estimated for individuals. Typical model simulations for various values of *δ_V_* and *N_max_* are presented in **Figure** 2 **.** We include a short simulation study in **Supplementary Material 1** that demonstrates the benefit of rich datasets for parameter identifiability. Our study showed that there is a large uncertainty associated with the parameter *N_max_*. The mathematical explanation for the uncertainty is that as $N\to \infty ,\alpha_{T}T\left(1-\frac{T}{NV}\right)\to \alpha_{T}T$. Therefore, large estimates for *N_max_* will give similar simulation results.

### Modeling techniques

Our nonlinear mixed‐effects model was implemented using Monolix software, which allows estimation of population parameters, interindividual variability (IIV) via the Stochastic Approximation to Expectation Maximization (SAEM) algorithm, and also individual parameters.^35^

For mixed‐effects modeling, it is assumed that the observed data, *y*, can be represented as a function of population and individual parameters and the experimental error on measurements. The structural model, *f*, describes the deterministic processes that give rise to the data and depends on time and the underlying model parameters. The error model, *g*, describes how measurement errors made during data collection change over time and their dependence on underlying parameters. Measurement *j* for individual *i* may be written:

(6) $$y_{ij}=f(t_{ij},\phi_{i})+g(t_{ij},\phi_{i})\epsilon_{ij},$$

where the vector $\phi_{i}$ contains the parameters corresponding to individual *i* for the structural model, *t_ij_* is the time of measurement *j* for individual *i*, and $\epsilon_{ij}$ is the residual error of the measurement.

We use a proportional error model defined by $g(t_{ij},\phi_{i})=bf(t_{ij},\phi_{i})$ where *b* is a positive constant. For our model, $\phi_{i}$ contains the parameters $\alpha_{V},\alpha_{T},K,\delta_{V},N_{\text{max}},t_{norm1},t_{norm2}$ and *b*, the error model parameter, the size of *y* is 30 × 10 (total number of individuals × number of measurements per individual). The observed and simulated data *y* and *f*, respectively, are tumor size measurements for 30 animals at 10 time points.

Model selection is performed by comparing the Bayesian Information Criterion (BIC) for each model, alongside visual predictive checks (VPC) and residual standard error (RSE) of population parameters and IIV. The BIC is a penalized likelihood criterion calculated by the formula BIC= $-2LL_{y}(\theta )+log(n)d$, where LL is the log‐likelihood, *n* is the number of observations, *θ* is the vector containing the population parameters, and *d* is the total number of parameters.

## RESULTS

### A simple monotonic vessel inhibition model does not capture transient dynamics

**Figure 3:**
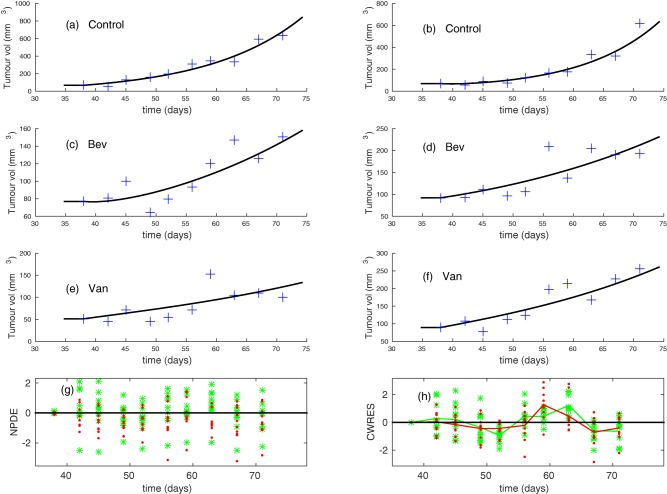
Monotonic model results from simulations of Eqs. 3**–**5 where *N_max_* = 1. (**a**)‐(**f**) Typical individual fits selected at random for (**a,b**) control, (**c,d**) bevacizumab, and (**e,f**) vanucizumab groups using experimental data. Blue **“**+**”**: experimental data; solid black lines: predicted tumor volume, *T*, using individual parameters estimated by Monolix. (**g**) Experimental (observed) results for tumor volume plotted against predicted results for tumor volume for individuals and colored by group. (**h**) Conditional weighted residuals (CWRES) plots for the transient dynamics model. The mean CWRES for each experimental group is shown by a solid line of the corresponding color. Blue circles: control; green stars: bevacizumab; red dots: vanucizumab. The plots show that the tumor volume is consistently underestimated for the treatment groups at day 52 and overestimated at days 59 and 63.

Model simulations that account for drug‐induced vessel regression, but not normalization (see Eqs. (3), (4) with *N_max_* = 1), produced a poor fit to the experimental data; the fit could be improved by accounting for the transient dynamics of tumor growth. The individual fits and residuals are presented in conditional weighted residuals in **Figure** 3. From **Figure** 3 **h**, it is clear that the tumor volume is almost always underestimated at day 52, and almost always overestimated at days 59 and 63. This is due to a steep increase in the tumor growth rate between these times. In the next subsection we improve the model by accounting for the transient tumor growth dynamics. The BIC for the monotonic model without normalization is 2,759. The results for the model parameters estimated for the monotonic model are presented in **Table** 1.

**Table 1:** Population parameters and interindividual variability (IIV) for the monotonic (Eqs. (3–5) with *n* = 1 for all time) and final (Eqs. (3–5)) structural models

Parameter	Units	Description	Monotonic model population parameter (RSE%)	Monotonic model IIV (RSE%)	Monotonic model shrinkage (%)	Final model population parameter (RSE%)	Final model IIV (RSE%)	Final model shrinkage (%)
α_T_	day^−1^	Tumor growth rate	0.11 (25)	0.134 (274)	−12	0.109 (12)	0.422 (25)	−6
α_V_	day^−1^	Vessel growth rate	0.0886 (20)	0.345 (47)	24	0.119 (7)	0.177 (34)	6
K	dimensionless	Initial ratio of V/T	1.26 (20)	0.941 (16)	16	1.14 (15)	0.711 (15)	9
t_norm1_	days	Start time of normalization window	—	—		53.4 (4)	0.1 FIX	—
t_norm2_	days	End time of normalization window	—	—		59.2 (3)	0.1 FIX	—
N_max_	dimensionless	Maximum normalization constant	—	—		6.7 (27)	0.55 (41)	—
δ_V_ (bev)	day^−1^	Vessel kill rate (bevacizumab)	0.0633 (33)	0.674 (2.3e3)		0.113 (11)	0.208 (47)	—
δ_V_ (van)	day^−1^	Vessel kill rate (vanucizumab)	0.0733 (32)	0.219 (176)		0.115 (12)	0.293 (33)	—
b	%	Proportional error parameter	22.7 (5)	—		18.5 (5)	—	—

The IIV given is the standard deviation ω estimated using Monolix sofware and the relative standard error (RSE) is a measure of the precision of the parameter estimates. Shrinkage is a measure used to compare the distribution of the individual parameters to the population parameters and is calculated via the formula: $Shrinkage=1-\frac{Var(\hat{\eta })}{\omega^{2}}$ where $Var(\hat{\eta })$ is the variance of the estimated individual parameters and ω^2^ is the estimated variance of the population parameters. A proportional error model was used and the percentage error is listed as parameter b. Parameters marked “FIX”' were held at stated values, and not estimated.

### Model selection: identifying the transient window of enhanced tumor growth

Following ref. 12, we assume that the duration of the transiently enhanced tumor growth window is similar for all animals injected with a given tumor cell line and receiving a particular antiangiogenic treatment. Therefore, in our model selection process we fix the variance of the population distributions for *t_norm_*_1_ and *t_norm_*_2_ to 0.1 and allow individual parameter values to be chosen within this predefined distribution. Since we assume that the transient window of enhanced tumor growth is caused by vessel normalization, we base further assumptions on experiments from ref. 13, where the time frame in which vessel normalization occurred was similar for all animals treated with bevacizumab. Based on experiments in refs. 13 and 12, we assume that the normalization constant remains at control levels before and after the transient window.

When assuming the antiangiogenic treatment stimulates both vessel regression and normalization, Eqs. 3–5 were used to estimate population and individual parameter values. During model selection, we tested several assumptions regarding the treatment parameters $\delta_{V},N_{\text{max}},t_{norm1}$, and *t_norm_*_2_ (see **Supplementary Material 2**). The model that gave the best fit to the experimental data assumed different values of *δ_V_* for both treatment groups, and the same values of $N_{\text{max}},t_{norm1}$, and *t_norm_*_2_ for both treatment groups. The parameter estimates, along with their RSE values and shrinkage, are listed in **Table** 1.

**Figure 4:**
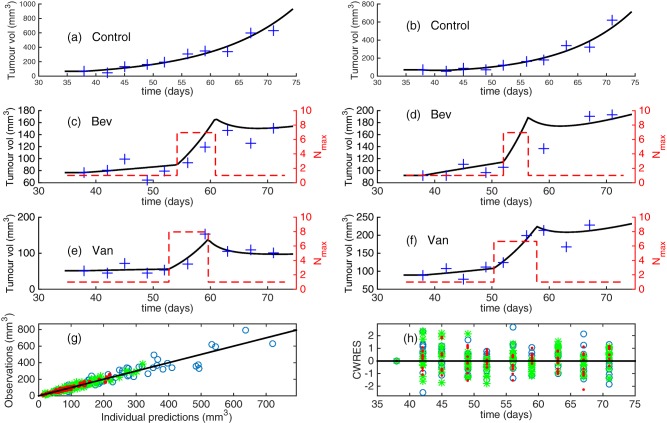
Transient dynamics model results from simulations of Eqs. (3**–**5). (**a–f**) Typical individual fits selected at random for (**a,b**) control; (**c,d**) bevacizumab, and (**e,f**) vanucizumab groups using experimental data. Blue **“**+**”**: experimental data; solid black lines, predicted tumor volume, *T*; red dashed lines, normalization windows for individuals. (**g**) Experimental (observed) results for tumor volume plotted against predicted results for tumor volume for individuals and colored by group. (**h**) Conditional weighted residuals (CWRES) plots for the transient dynamics model. Key: blue circles: control; green stars: bevacizumab; red dots: vanucizumab.

**Figure 5:**
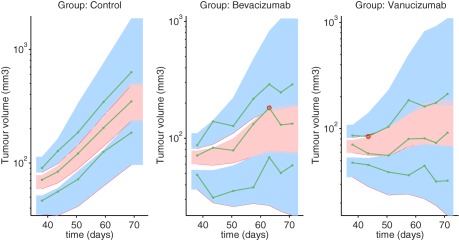
Visual predictive check (VPC) for the final model (Eqs. 3**–**5) split by group. Parameters of the final model are listed in **Table 1**. Green, solid lines show the 10%, 50%, and 90% quantiles of the observed data and the shaded regions represent the 90% prediction intervals on the theoretical 10% (blue region, lower), 50% (red region, middle), and 90% (blue region, upper) quantiles. Outliers are highlighted by red circles.

The individual fits and residual errors for the final model are shown in **Figure** 4 and the visual predictive checks (VPCs), split by experimental group, are shown in **Figure** 5. These results show that the transient dynamics model (Eqs. (3), (4), (5)) describes individual and population data well for all groups. We performed a likelihood ratio test (LRT) and found that the results agreed with the Wald test that the data are better described using a proportional error model than a constant error model ( −Δ2LL=−32764.61).

We selected the transient dynamics model as the most appropriate to represent the experimental data based on the diagnostic plots, shrinkage, parameter estimates, and RSE values, compared to the monotonic model (Eqs. 3–5 with *N_max_* = 1). The BIC for the transient dynamics model, *BIC_T_*, was larger than the BIC for the monotonic model, *BIC_M_*, ( $BIC_{T}=2865,BIC_{M}=2797$). However, we do not reject the transient dynamics model based on the BIC, and we propose that the other evidence for model appropriateness (diagnostic plots, RSE, shrinkage) suggests that the normalization model best describes the data.

The estimated value of the population parameter *N_max_* = 6.7. An interpretation of this result is that, during the transient window, the vasculature provides sufficient oxygen and nutrients to support a tumor 6.7 times larger than it was able to support before the period of enhanced tumor growth.

### Verification with histology data

We compared the histology results for vessel density (measured in vessels per mm^2^ tumor tissue) at day 71 with the simulated results for vessel density (*V*/*T*) at day 71. Details of the comparison are given in **Supplementary Material 4**. We observed that there is good qualitative agreement between the histology and the simulated data.

### Theoretical administration of chemotherapy

**Figure 6:**
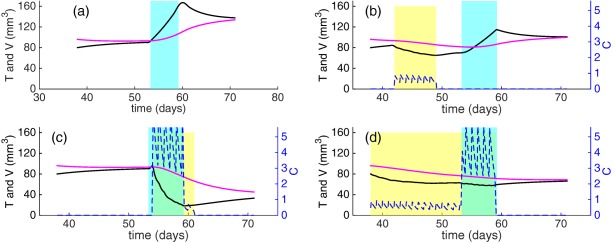
Model simulations for antiangiogenic monotherapy, and three alternative theoretical treatment regimens to combine antiangiogenic therapy and chemotherapy, Eqs. 7**–**9. Key: Black lines: tumor volume (*T*), magenta lines: vessel‐dependent carrying capacity (*V*); dashed blue lines: intratumoural concentration of the cytotoxic drug, *C*. Blue shaded regions represent the normalization window and yellow shaded regions represent the delivery period of chemotherapy. (**a**) No chemotherapy, (**b**) chemotherapy administered before the transient window of enhanced tumor growth, (**c**) chemotherapy administered during the transient window of enhanced tumor growth, (**d**) chemotherapy administered for a three week period from day 38 to day 59. Antiangiogenic therapy is administered from day 38 as in the preclinical study.

We now simulate the administration of a cytotoxic drug, *C*(*t*), in order to examine whether the model predicts a more pronounced difference in tumor volume when chemotherapy is administered during the normalization window. We assume that the cytotoxic drug is delivered to the tumor at a rate proportional to *N* × *V*, and that it acts to kill tumor cells at a rate proportional to its concentration in the tumor. The chemotherapy model is based on the model proposed previously,^28^ which investigates the effects of docetaxel and capecitabine on tumor growth. For simplicity, we based our parameter estimates on the population parameters from this model. The equations for vascular tumor growth in response to combined antiangiogenic and chemotherapy are given by:

(7) $$\frac{dT}{dt}=\alpha_{T}\;T\left(1-\frac{T}{N\times V}\right)\;-\;\delta_{T}\;C\;Te^{-\lambda t},$$

(8) $$\frac{dV}{dt}=\alpha_{V}\;T^{2/3}\;V^{1/3}-\delta_{V}\;V,$$

(9) $$C=\alpha_{C}(t)NVe^{-k\;\text{mod}(t,1)},$$

(10) $$N(t)=\{\begin{array}{cc} 1 & \text{for}\;t\leq t_{norm1}\;\text{and}\;t\geq t_{norm2},\\ N_{\text{max}} & \text{for}\;t_{norm1}<t<t_{norm2}, \end{array}$$

and

(11) $$\alpha_{C}(t)=\{\begin{array}{cc} 0 & \text{for}\;t\leq t_{Con}\;\text{and}\;t\geq t_{Coff},\\ {\tilde{\alpha }}_{C} & \text{for}\;t_{Con}<t<t_{Coff} \end{array}$$

where *C* is the concentration of the cytotoxic drug in mg ml^–^ 1 inside the tumor. We use the population values for the model parameters in response to vanucizumab, and fix the new parameters so that ${\tilde{\alpha }}_{C}=1\;{\text{mg}\;\text{ml}}^{-1}{\text{mm}}^{-3},k=0.9{\;\text{day}}^{-1},\;\delta_{T}=0.12{\;\text{mg}}^{-1}{\text{ml}\;\text{day}}^{-1},\lambda =0.08{\;\text{day}}^{-1}$. We consider three situations. First, chemotherapy is administered once daily for a 1‐week period before the transient window, so that $\left(t_{Con},t_{Coff})=(42,49\right)$; in the second case, chemotherapy is administered once daily for 1 week during the transient window, so that $\left(t_{Con},t_{Coff})=(54,61\right)$; and in the third case, chemotherapy is administered for a 3‐week period that starts on the same day as antiangiogenic therapy, so that $\left(t_{Con},t_{Coff})=(38,59\right)$. The third case is likely to be the most realistic regimen administered to patients, and represents treatment when the timing of the normalization window is not known. The results of our simulations are shown in **Figure** 6. When chemotherapy is administered before the transient window, tumor growth is reduced, but chemotherapy is more efficacious when administered during the transient window, leading to a more pronounced decrease in tumor volume. Interestingly, our model predicts that chemotherapy administered both before and during the normalization window (**Figure** 6 **d**) leads to a smaller reduction in tumor volume than chemotherapy administered only within the normalization window.

The simulations in this section are based on the assumption that resistance to chemotherapy emerges. The resistance term, $e^{-\lambda t}$, in Eq. (7) reduces the efficacy of the chemotherapy agent at long times, and this is likely to influence the reduction in tumor volume in **Figure** 6 **d**, compared to **Figure** 6 **c**. We show that changes in the resistance parameter *λ* or ±20% do not change the conclusions in this section in **Supplementary Material 5**.

## DISCUSSION

To our knowledge, this is the first semimechanistic mixed‐effects model that accounts explicitly for the effects of vessel normalization in response to antiangiogenic therapy. Our model was motivated through the identification of transient dynamics in the experimental data (data shown in **Figure** 1), and builds upon recent, similar models.^27^, ^31^ by incorporation of mathematical representations for the transient tumor growth dynamics. Based on our results, we conclude that mixed‐effects modeling can be used to locate and parameterize the window of enhanced tumor growth, which may be a direct or indirect effect of the vessel normalization window, for KPL‐4 xenografts, leveraging only tumor size data. In addition, our model predicts that cytotoxic therapies could lead to a greater decrease in tumor volume if administered within the transient window. Our model allows us to quantify synergism between chemotherapy and antiangiogenic therapy, given the hypothesis that the delivery of chemotherapy is enhanced during the transient window. The experimental design could be improved to minimize the RSE of estimated parameters, for example by measuring the tumor volume via imaging methods instead of caliper methods.

We hypothesize that the transient window that we identify from our experimental data can be attributed to multiple processes, which include increased pericyte coverage, increased vessel perfusion, and decreased vessel permeability (leakiness). These physiological variables are assumed directly to increase tumor oxygenation and, indirectly, increase efficacy of chemotherapy and radiotherapy. Techniques such as window chamber assays and fluorescent staining are available to measure such physiological variables *in vivo*. The next step of model validation would involve performing experiments that can measure dynamic vessel volume to investigate whether the window of enhanced tumor growth that we identify corresponds to the above aspects of vessel normalization, and whether chemotherapy is more efficacious when administered within the window.

Experiments performed on mouse xenografts suggest that normalization can occur 3 days after the onset of treatment.^4^, ^12^, ^13^, ^36^ The transient window that we identified begins 15 days after the start of antiangiogenic treatment. It is possible that the enhanced tumor growth period that we identified is a downstream effect of vessel normalization, and that normalization begins earlier. Further experiments are required to resolve this discrepancy.

Previous experiments have shown that, during the transient normalization window, the efficacy of radiotherapy and chemotherapy are enhanced.^4^, ^5^, ^12^, ^13^ If the window is not taken into account when investigating the efficacy of combined antiangiogenic therapy with radiotherapy or chemotherapy, then inconsistencies in efficacy measurements may result. With validation, our model has the potential to provide a thorough understanding of the likely effect on efficacy measurements that the changing vasculature may have.

We developed and parameterized the model using longitudinal tumor size data in a single preclinical tumor model, and the dynamic carrying capacity was inferred. The scope of our semi‐mechanistic model is limited by the quality, quantity, and type of available experimental data. A pooled approach was used for parameter estimation, to maximize the amount of data used to estimate the tumor and vessel growth parameters. For the final model, we fixed the IIV of *t_norm_*_1_ and *t_norm_*_2_ to 0.1 in order to allow small variations in the estimates for the start and end times of the transient window. No other parameter values were manipulated or fixed, except for the parameters for the chemotherapy simulations.

The results for the vascular volume after treatment were in qualitative agreement with histology data, and the inconsistency in the results for vessel density in control groups can be explained via a plausable argument regarding intra‐ and extra‐tumoral blood vessels.

Our model could be used to identify the transient window associated with other antiangiogenic treatments and tumor cell lines, in both preclinical and clinical settings. In addition, our model could be used to identify the optimal time for combination treatment, especially given the experimental observations in refs. 12 and 13 suggest that combination therapies in which radiotherapy or chemotherapy are administered during the normalization window achieve better outcomes than when administered before or after the window.

## Supporting information

Supporting Information

Supporting Information

Supporting Information

Supporting Information

Supporting Information

Supporting Information
