# Supplementary material for: Modeling Longitudinal Preclinical Tumor Size Data to Identify Transient Dynamics in Tumor Response to Antiangiogenic Drugs
Source: CPT Pharmacometrics Syst Pharmacol. 2016 Nov 14;5(11):636–45. doi: 10.1002/psp4.12142 (PMC5192995; doi:10.1002/psp4.12142)
Supplement: Supplementary file 1 — Supporting Information [file PSP4-5-636-s001.pdf]

Modelling longitudinal preclinical tumour size data to identify transient dynamics in tumour response to anti-angiogenic drugs

L G Hutchinson [a], H-J Mueller [d], E A Gaffney [a], P K Maini [a], J Wagg [b], A Phipps [c], C Boetsch [b], H M Byrne [a], B Ribba [b]

August 22, 2016

a. Wolfson Centre for Mathematical Biology, Mathematical Institute, University of Oxford, Andrew Wiles Building, Radcliffe Observatory Quarter, Woodstock Road, Oxford OX2 6GG, UK

b. Roche Pharmaceutical Research & Early Development, Roche Innovation Center Basel, Switzerland

c. Pharma Research and Early Development, Roche Innovation Centre Welwyn, 6 Falcon Way, Shire Park, Welwyn Garden City, AL7 1TW, UK

d. Pharma Research and Early Development, Roche Innovation Centre Munich, Nonnenwald, 82377, Penzberg, Germany

Corresponding author: Lucy Grace Hutchinson: hutchinson@maths.ox.ac.uk
+44 1865 283 874

Keywords: angiogenesis; normalization; modelling and simulation; tumour size; preclinical

Tables: 1, Figures: 6

Word counts:
Abstract 150
Main body 4004

Abstract

Experimental evidence suggests that anti-angiogenic therapy gives rise to a transient window of vessel normalization, within which, the efficacy of radiotherapy and chemotherapy may be enhanced. Preclinical experiments that measure components of vessel normalization are invasive and expensive. We have developed a mathematical model of vascular tumour growth from preclinical time-course data in a breast cancer xenograft model. We used a mixed effects approach for model parameterisation, leveraging tumour size data to identify a period of enhanced tumour growth that could potentially correspond to the transient window of vessel normalization. We estimated the characteristics of the window for mice treated with an anti-VEGF antibody (bevacizumab) or with a bispecific anti-VEGF/anti-angiopoietin-2 antibody (vanucizumab). We show how the mathematical model could theoretically be used to predict how to coordinate anti-angiogenic therapy with radiotherapy or chemotherapy to maximise therapeutic effect, reducing the need for preclinical experiments that directly measure vessel normalization parameters.

1 Introduction

Angiogenesis is the process by which blood vessels form from existing ones; it plays a key role in tumour growth and progression. The initial development of anti-angiogenic therapies was based on the premise that pruning new tumour vessels would reduce the blood supply to the tumour, and inhibit the delivery of oxygen and nutrients to the tumour, causing its growth to slow down or stop [1]. However, it is now clear that anti-angiogenic therapy not only causes vascular regression, but also affects processes including vessel permeability, perfusion, diameter, tortuosity and pericyte coverage; and thereby normalizes the vasculature [2]. It has been suggested that vessel normalization plays a key role in tumour progression, since it may transiently enhance the delivery of oxygen and nutrients to the tumour microenvironment [3]. There is evidence that anti-angiogenic drug induced vessel normalization transiently increases the efficacy of chemotherapy and radiotherapy [4, 5]. If this normalization window were identified for individual patients, then combination treatment schedules could be designed in which administration of chemotherapy or radiotherapy would be coordinated with the normalization window to maximise the therapeutic response.

Normalization has been observed in preclinical models of anti-angiogenic therapy both from histology [6, 7] and via real-time imaging methods such as window chamber assays [8, 9]. For reviews on the role of normalization in neovascular development, see [10] and [11]. Evidence from mouse xenograft studies suggests that vessel normalization is a transient effect that begins shortly after the onset of anti-angiogenic therapy and ends a few days later [12, 13]. Furthermore, some clinical studies are consistent with anti-angiogenic therapy stimulating a reduction in vessel permeability for glioblastoma [14] and rectal cancer [15]. In both [12] and [13] radiotherapy was found to be most efficacious when administered within the transient window of increased tumour oxygenation. In particular, in [12] a synergistic tumour growth delay was observed when radiotherapy was administered 4-6 days after the first dose of anti-angiogenic therapy. It has also been suggested that normalized vessels allow efficient delivery of chemotherapy since improved perfusion allows effective extravasation of small molecules [5, 16].

Bevacizumab is an anti-vascular endothelial growth factor (VEGF) antibody that has been approved for treatment of numerous cancers including renal cell carcinoma, non-small-cell lung cancer and colorectal cancer. Vanucizumab is a bispecific antibody that

recognises VEGF with one arm (based on bevacizumab) and angiopoietin-2 (Ang2) with the other arm (based on LC06, an Ang2 specific antibody). Vanucizumab is currently in phase 2 trials to treat locally advanced or metastatic solid tumours (NCT01688206), and in combination with FOLFOX for metastatic colorectal cancer (NCT02141295). It is thought that since both VEGF and Ang2 promote angiogenesis, simultaneous inhibition of the ligands will result in an additive or even synergistic effect on tumour vessel regression [7]. Anti-angiogenic therapies are principally used in combination with cytotoxic therapies or radiotherapy. It has been shown that the timing of such concomitant treatments is important for the therapeutic outcome in preclinical experiments [4, 5]. In order to further elucidate some of the mechanisms by which anti-angiogenic therapy can improve treatment outcome when administered alone or in combination with chemotherapy, we have developed a mathematical model of vascular tumour growth. Our model can be used to identify a period of enhanced tumour growth that could correspond to the vessel normalization window within which cytotoxic or radiotherapeutic efficacy may be increased.

The development of mathematical models of angiogenesis has been driven by experimental results. Continuous, discrete and hybrid models can be used to represent vessel growth dynamics, and tumour growth, in one, two or three dimensions and may account for intricate biological details [17, 18, 19, 20, 21] (for a review, see [22]). Without suitable experimental data, these models can yield qualitative mechanistic insight; with appropriate data, the models can be validated and parameterised and, thereby, their predictive power increased. Mathematical models canalso be used to investigate the impact of mechanistic perturbations to angiogenesis and to formulate hypotheses about optimal therapy regimens.

For model development and parameterisation, non-linear mixed effects (NLME) modelling enables a data-driven approach [23]. A maximum likelihood approach is used to estimate population and individual parameters from experimental data. The method has been used widely to integrate tumour growth data with ordinary differential equation (ODE) models that characterise tumour growth kinetics in the presence and absence of cytotoxic treatments [24, 25, 26, 27, 28]. Typically these models comprise a term that represents an empirical tumour growth law (eg. logistic, Gompertzian, or exponential growth), and another term to represent tumour growth inhibition due to chemotherapy. For example, in [24] a two-phase tumour growth law (exponential followed by linear) is

modified to account for a cytotoxic therapy which acts directly to kill the tumour cells. This model is extended in [29] to account for anti-angiogenic therapy which is assumed to indirectly slow the tumour's growth rate, however, there is no variable for the tumour's vascular density.

Building on these models, Hahnfeldt *et al.* proposed a simple model of vascular tumour growth in which the tumour and the vasculature are treated separately. Vascular density is assumed to regulate the equilibrium size of the tumour while the tumour is assumed to promote angiogenesis [30]. In [31], Ouerdani *et al.* develop a model of vascular tumour growth in which a logistic tumour growth law is assumed, and the equilibrium tumour size is the vessel-dependent carrying capacity. The authors use preclinical and clinical data to parameterise the model in the presence and absence of the dual cytotoxic and anti-angiogenic drug, pazopanib. Similarly, in [27], Wilson *et al.* present a logistic tumour growth model with a dynamic carrying capacity term that is parameterised for administration of anti-angiogenic therapy alone and in combination with chemotherapy. The authors use their model to predict an optimal time for the administration of chemotherapy following administration of anti-angiogenic therapy. They suggest that vascular normalization could play a key role in the identification of the optimal treatment schedule, although normalization is neglected in their model.

It is clear that vessel normalization plays a key role in vascular tumour growth, although parameters associated with normalization are rarely measured. While existing mixed effects models have not explicitly considered the dynamics of vessel normalization, in this paper, we are motivated by the transient dynamics apparent from the experimental data in the KPL-4 preclinical mouse (xenograft) model of breast cancer to extend existing mathematical models of vascular tumour growth under anti-angiogenic therapy to account for these dynamics. Our primary goal is to combine mixed effects modelling with tumour size data from KPL-4 mouse xenografts to characterise the transient window of increased tumour growth following exposure to anti-angiogenic therapy. The same model could be used to characterise normalization in clinical studies. To conclude our study, we demonstrate how the model can be used to predict the optimal time, relative to the transient window, to schedule chemotherapy.

2 Methods

2.1 Preclinical data

Female SCID beige mice, aged 8 weeks, were housed in specific-pathogen-free conditions according to committed guidelines (GV-Solas, Felasa, TierschG) and injected with a suspension of 3×10^6 KPL-4 tumour cells into the right, penultimate, inguinal mammary fat pad. Treatment started 38 days after tumour cell injection, when tumours had reached a mean size of 70mm^3, and mice were randomized into control (omalizumab), bevacizumab treatment and vanucizumab treatment groups with 10 mice per group.

Bevacizumab is an anti-VEGF antibody, vanucizumab is a bispecific anti-VEGF/anti-Ang2 antibody that neutralises both ligands, and omalizumab was included as an isotype control. Each antibody was administered via i.v. injection at a dose of 10mg/kg, once per week, starting 38 days after inoculation for a total of 5 weeks.

Tumour volume, T, was calculated using the formula $T = \frac{\text{length} \times \text{width}^2}{2}$ where the length and width of the tumour were the longest and shortest dimensions of the tumour lying at 90° to the longest, respectively. These measurements were taken twice per week for the treated period, resulting in a total of 10 tumour size measurements per animal. The data are presented in Figure 1.

2.2 Model development

Untreated tumours

The structural model that we present comprises two ordinary differential equations (ODEs) to describe time dependent tumour growth and associated vessel-dependent carrying capacity and is inspired by a model devised by Hahnfeldt *et al.* [30]. The tumour size is the observed variable; the vessel-dependent carrying capacity has not been measured experimentally. We assume that all individual parameters are distributed log-normally, which is generally accepted for growth rates and reaction rates [32].

The biological interpretation of the carrying capacity is the maximum tumour size that can be supported by the associated vasculature. The anti-angiogenic therapies that we consider affect vessel growth, but are not directly cytotoxic. Therefore, we view tumour volume, T (measured in mm^3), and the carrying capacity, V (also in mm^3), as distinct dependent variables. A simple logistic growth model for T was chosen for the untreated

case (see equation (1)). Tumour growth has been represented using a logistic or generalised logistic growth term in several related models [31, 27, 25]. Following [30], we assume that the carrying capacity, V, of the tumour depends on the local vascular density and architecture. In recent publications [27, 31], the evolution of the carrying capacity was assumed to depend only on T. We propose that it is realistic to take into consideration the existing vascular density since new vessels sprout from existing ones. The untreated model of tumour size and dynamic carrying capacity can be written:

$$\frac{\mathrm{d}T}{\mathrm{d}t} = \alpha_T \, T \left(1 - \frac{T}{V}\right), \tag{1}$$

$$\frac{\mathrm{d}V}{\mathrm{d}t} = \alpha_V \, T^{\beta} \, V^{\gamma}, \tag{2}$$

where α_T is the maximal tumour growth rate, and α_V is the growth rate of the vascular-dependent carrying capacity. Since in equation (2) the exponents β and γ are not identifiable via model simulations, we fixed them at physiologically based values. Vessel growth is stimulated by growth factors (such as VEGF) that are released by tumour cells in response to hypoxia and, by a simple geometrical argument, we may assume that the proportion of the tumour volume that is hypoxic is proportional to its surface area. This assumption is consistent with the pO2 gradient in tumours described in [33]. Accordingly, we fix $\beta = \frac{2}{3}$. We use a value of $\gamma = 1 - \beta = \frac{1}{3}$ to ensure that vessel growth is exponential at long times. We note that there are several values for β and γ that could feasibly represent vessel dependent tumour growth: the values chosen here are based on geometrical arguments.

Tumours treated with anti-angiogenic therapy

We assume that anti-angiogenic therapy has two effects: (1) it causes blood vessel regression and (2) it leads to a transient period during which tumour growth is enhanced due to increased blood flow. We start by taking only effect (1) into account, and then suppose both effects are active. We incorporate effects (1) and (2) into our mathematical model by including a term for vessel regression and a function that transiently increases the carrying capacity (this change being stimulated by increased tumour perfusion during the transient normalization window).

In the absence of pharmacokinetic data from the experiment and to avoid introducing further unknown model parameters, we do not explicitly model drug levels in the tumour microenvironment; we assume that from the onset of treatment, the concentration of each

anti-angiogenic drug in the blood stream is maintained at a high enough level to ensure maximum efficacy. The long half-life (around 8 days in preclinical models) of bevacizumab supports this assumption [34], and we assume that vanucizumab has a similarly long half-life. Data presented by [34] and [7], shows that a weekly dose of 10mg/kg of bevacizumab or vanucizumab results in maximum efficacy with respect to tumour growth inhibition in mice, further supporting our argument. The full model can be written

$$\frac{\mathrm{d}T}{\mathrm{d}t} = \alpha_T \, T \left(1 - \frac{T}{N \times V}\right), \tag{3}$$

$$\frac{\mathrm{d}V}{\mathrm{d}t} = \alpha_V \, T^{2/3} \, V^{1/3} - \delta_V \, V, \tag{4}$$

$$N(t) = \begin{cases} 1 \text{ for } t \leq t_{norm1} \text{ and } t \geq t_{norm2}, \\ N_{max} \text{ for } t_{norm1} < t < t_{norm2}, \end{cases} \tag{5}$$

where the constant δ_V corresponds to the vessel death rate, t_{norm1} and t_{norm2} are the start and end times of the window of enhanced tumour growth, respectively, and $N_{max} \geq 1$ is the maximum factor by which the carrying capacity is enhanced during the transient window. For the first case, where only effect (1) is accounted for, $N_{max} = 1$ for all time.

The initial condition for T is the experimental value of the tumour volume at the first measurement time (day 38). We estimate the initial value of V via the parameter $K = \frac{T_0}{V_0}$, where T_0 is the observed initial tumour volume (observed) and V_0 is the initial vessel dependent carrying capacity. As such we assume that V_0 is linearly related to T_0 for individuals through the parameter K, which is estimated for individuals. Typical model simulations for various values of δ_V and N_{max} are presented in Figure 2. We include a short simulation study in Supplementary Material 1 that demonstrates the benefit of rich datasets for parameter identifiability. Our study showed that there is a large uncertainty associated with the parameter N_{max}. The mathematical explanation for the uncertainty is that as $N \to \infty$, $\alpha_T T \left(1 - \frac{T}{NV}\right) \to \alpha_T T$.Therefore large estimates for N_{max} will give similar simulation results.

2.3 Modelling Techniques

Our non-linear mixed effects model was implemented using Monolix software, which allows estimation of population parameters, inter-individual variability (IIV) via the Stochastic Approximation to Expectation Maximisation (SAEM) algorithm, and also individual pa-

rameters [35].

For mixed effects modelling, it is assumed that the observed data, y, can be represented as a function of population and individual parameters and the experimental error on measurements. The structural model, f, describes the deterministic processes that give rise to the data and depends on time and the underlying model parameters. The error model, g, describes how measurement errors made during data collection change over time and their dependence on underlying parameters. Measurement j for individual i may be written

$$y_{ij} = f(t_{ij}, \phi_i) + g(t_{ij}, \phi_i)\epsilon_{ij}, \tag{6}$$

where the vector ϕ_i contains the parameters corresponding to individual i for the structural model, t_{ij} is the time of measurement j for individual i, and ϵ_{ij} is the residual error of the measurement.

We use a proportional error model defined by $g(t_{ij}, \phi_i) = bf(t_{ij}, \phi_i)$ where b is a positive constant. For our model, ϕ_i contains the parameters $\alpha_V, \alpha_T, K, \delta_V, N_{max}, t_{norm1}, t_{norm2}$ and b, the error model parameter, the size of y is 30×10 (total number of individuals$\times$ number of measurements per individual). The observed and simulated data y and f, respectively, are tumour size measurements for 30 animals at 10 time points.

Model selection is performed by comparing the Bayesian Information Criterion (BIC) for each model, alongside visual predictive checks (VPC) and residual standard error (r.s.e.) of population parameters and IIV. The BIC is a penalized likelihood criterion calculated by the formula BIC $= -2\mathcal{LL}_y(\theta) + log(n)d$, where $\mathcal{LL}$ is the log-likelihood, n is the number of observations, θ is the vector containing the population parameters and d is the total number of parameters.

3 Results

3.1 A simple monotonic vessel inhibition model does not capture transient dynamics

Model simulations that account for drug-induced vessel regression, but not normalization (see equations (3)-(4) with $N_{max} = 1$), produced a poor fit to the experimental data; the fit could be improved by accounting for the transient dynamics of tumour growth. The individual fits and residuals are presented in conditional weighted residuals in Figure 3.

From Figure 3(h), it is clear that the tumour volume is almost always under-estimated at day 52, and almost always over-estimated at days 59 and 63. This is due to a steep increase in the tumour growth rate between these times. In the next subsection we improve the model by accounting for the transient tumour growth dynamics. The BIC for the monotonic model without normalization is 2759. The results for the model parameters estimated for the monotonic model are presented in Table 1.

3.2 Model selection: identifying the transient window of enhanced tumour growth

Following [12], we assume that the duration of the transiently enhanced tumour growth window is similar for all animals injected with a given tumour cell line and receiving a particular anti-angiogenic treatment. Therefore, in our model selection process, we fix the variance of the population distributions for t_{norm1} and t_{norm2} to 0.1 and allow individual parameter values to be chosen within this pre-defined distribution. Since we assume that the transient window of enhanced tumour growth is caused by vessel normalization, we base further assumptions on experiments from [13], where the time frame in which vessel normalization occurred was similar for all animals treated with bevacizumab. Based on experiments in [13] and [12], we assume that the normalization constant remains at control levels before and after the transient window.

When assuming the anti-angiogenic treatment stimulates both vessel regression and normalization, equations (3)-(5) were used to estimate population and individual parameter values. During model selection, we tested several assumptions regarding the treatment parameters $\delta_V, N_{max}, t_{norm1}$ and t_{norm2} (see Supplementary Material 2). The model that gave the best fit to the experimental data assumed different values of δ_V for both treatment groups, and the same values of N_{max}, t_{norm1} and t_{norm2} for both treatment groups. The parameter estimates along with their r.s.e. values and shrinkage are listed in Table 1.

The individual fits and residual errors for the final model are shown in Figure 4 and the visual predictive checks (VPCs), split by experimental group, are shown in Figure 5. These results show that the transient dynamics model (equations (3)-(5)) describes individual and population data well for all groups. We performed a likelihood ratio test (LRT) and found that the results agreed with the Wald test that the data are better described using

a proportional error model than a constant error model ($-\Delta 2\mathcal{LL} = -32764.61$).

We selected the transient dynamics model as the most appropriate to represent the experimental data based on the diagnostic plots, shrinkage, parameter estimates and r.s.e. values, compared to the monotonic model (equations (3)-(5) with $N_{max} = 1$). The BIC for the transient dynamics model, BIC_T, was larger than the BIC for the monotonic model, BIC_M, ($BIC_T = 2865, BIC_M = 2797$). However, we do not reject the transient dynamics model based on the BIC, and we propose that the other evidence for model appropriateness (diagnostic plots, r.s.e., shrinkage) suggests that the normalization model describes the data the best.

The estimated value of the population parameter $N_{max} = 6.7$. An interpretation of this result is that, during the transient window, the vasculature provides sufficient oxygen and nutrients to support a tumour 6.7 times larger than it was able to support before the period of enhanced tumour growth.

3.3 Verification with histology data

We compared the histology results for vessel density (measured in vessels per mm^2 tumour tissue) at day 71 with the simulated results for vessel density (V/T) at day 71. Details of the comparison are given in Supplementary Material 4. We observed that there is good qualitative agreement between the histology and the simulated data.

3.4 Theoretical administration of chemotherapy

We now simulate the administration of a cytotoxic drug, $C(t)$ in order to examine whether the model predicts a more pronounced difference in tumour volume when chemotherapy is administered during the normalization window. We assume that the cytotoxic drug is delivered to the tumour at a rate proportional to $N \times V$, and that it acts to kill tumour cells at a rate proportional to its concentration in the tumour. The chemotherapy model is based on the model proposed in [28], which investigates the effects of docetaxel and capecitabine on tumour growth. For simplicity, we based our parameter estimates on the population parameters from this model. The equations for vascular tumour growth in response to combined anti-angiogenic and chemotherapy are given by:

$$\frac{\mathrm{d}T}{\mathrm{d}t} = \alpha_T\, T \left(1 - \frac{T}{N \times V}\right) - \delta_T\, C\, Te^{-\lambda t}, \tag{7}$$

$$\frac{\mathrm{d}V}{\mathrm{d}t} = \alpha_V\, T^{2/3}\, V^{1/3} - \delta_V\, V, \tag{8}$$

$$C = \alpha_C(t) N V e^{-k \bmod(t,1)}, \tag{9}$$

$$N(t) = \begin{cases} 1 \text{ for } t \leq t_{norm1} \text{ and } t \geq t_{norm2}, \\ N_{max} \text{ for } t_{norm1} < t < t_{norm2}, \end{cases} \tag{10}$$

and

$$\alpha_C(t) = \begin{cases} 0 \text{ for } t \leq t_{Con} \text{ and } t \geq t_{Coff}, \\ \tilde{\alpha}_C \text{ for } t_{Con} < t < t_{Coff} \end{cases} \tag{11}$$

where C is the concentration of the cytotoxic drug in mg ml^{-1} inside the tumour. We use the population values for the model parameters in response to vanucizumab, and fix the new parameters so that $\tilde{\alpha}_C = 1$mg ml^{-1}mm^{-3}, $k = 0.9$ day^{-1}, $\delta_T = 0.12$ mg^{-1}ml day^{-1}, $\lambda = 0.08$ day^{-1}. We consider three situations. First, chemotherapy is administered once daily for a one week period before the transient window, so that $(t_{Con}, t_{Coff}) = (42, 49)$; in the second case chemotherapy is administered once daily for one week during the transient window, so that $(t_{Con}, t_{Coff}) = (54, 61)$; and in the third case, chemotherapy is administered for a three-week period that starts on the same day as anti-angiogenic therapy, so that $(t_{Con}, t_{Coff}) = (38, 59)$. The third case is likely to be the most realistic regimen administered to patients, and represents treatment when the timing of the normalization window is not known. The results of our simulations are shown in Figure 6. When chemotherapy is administered before the transient window, tumour growth is reduced, but chemotherapy is more efficacious when administered during the transient window, leading to a more pronounced decrease in tumour volume. Interestingly, our model predicts that chemotherapy administered both before and during the normalization window (Figure 6(d)) leads to a smaller reduction in tumour volume than chemotherapy administered only within the normalization window.

The simulations in this section are based on the assumption that resistance to chemotherapy emerges. The resistance term, $e^{-\lambda t}$, in Equation 7 reduces the efficacy of the chemotherapy agent at long times, and this is likely to influence the reduction in tumour volume in Figure 6(d) compared to Figure 6(c). We show that changes in the resistance

parameter λ or $\pm 20\%$ do not change the conclusions in this section in Supplementary Material 5.

4 Discussion

To our knowledge, this is the first semi-mechanistic mixed-effects model that accounts explicitly for the effects of vessel normalization in response to anti-angiogenic therapy. Our model was motivated through the identification of transient dynamics in the experimental data (data shown in Figure 1), and builds upon recent, similar models [27, 31] by incorporation of mathematical representations for the transient tumour growth dynamics. Based on our results we conclude that mixed effects modelling can be used to locate and parameterise the window of enhanced tumour growth, which may be a direct or indirect effect of the vessel normalization window, for KPL-4 xenografts, leveraging only tumour size data. In addition, our model predicts that cytotoxic therapies could lead to a greater decrease in tumour volume if administered within the transient window. Our model allows us to quantify synergism between chemotherapy and anti-angiogenic therapy given the hypothesis that the delivery of chemotherapy is enhanced during the transient window. The experimental design could be improved to minimise the r.s.e. of estimated parameters, for example by measuring the tumour volume via imaging methods instead of caliper methods.

We hypothesise that the transient window that we identify from our experimental data can be attributed to multiple processes, that include increased pericyte coverage, increased vessel perfusion and decreased vessel permeability (leakiness). These physiological variables are assumed directly to increase tumour oxygenation and, indirectly, increase efficacy of chemotherapy and radiotherapy. Techniques such as window chamber assays and fluorescent staining are available to measure such physiological variables *in-vivo*. The next step of model validation would involve performing experiments that can measure dynamic vessel volume to investigate whether the window of enhanced tumour growth that we identify corresponds to the above aspects of vessel normalization, and whether chemotherapy is more efficacious when administered within the window.

Experiments performed on mouse xenografts suggest that normalization can occur 3 days after the onset of treatment [36, 12, 13, 4]. The transient window that we identified begins 15 days after the start of anti-angiogenic treatment. It is possible that the

enhanced tumour growth period that we have identified is a downstream effect of vessel normalization, and that normalization begins earlier. Further experiments are required to resolve this discrepancy.

Previous experiments have shown that, during the transient normalization window, the efficacy of radiotherapy and chemotherapy are enhanced [5, 4, 12, 13]. If the window is not taken into account when investigating the efficacy of combined anti-angiogenic therapy with radiotherapy or chemotherapy, then inconsistencies in efficacy measurements may result. With validation, our model has the potential to provide a thorough understanding of the likely effect on efficacy measurements that the changing vasculature may have.

We developed and parameterised the model using longitudinal tumour size data in a single preclinical tumour model, and the dynamic carrying capacity was inferred. The scope of our semi-mechanistic model is limited by the quality, quantity and type of available experimental data. A pooled approach was used for parameter estimation, to maximise the amount of data used to estimate the tumour and vessel growth parameters. For the final model, we fixed the IIV of t_{norm1} and t_{norm2} to 0.1 in order to allow small variations in the estimates for the start and end times of the transient window. No other parameter values were manipulated or fixed, except for the parameters for the chemotherapy simulations.

The results for the vascular volume after treatment were in qualitative agreement with histology data, and the inconsistency in the results for vessel density in control groups can be explained via a plausable argument regarding intra-and extra-tumoural blood vessels.

Our model could be used to identify the transient window associated with other anti-angiogenic treatments and tumour cell lines, in both preclinical and clinical settings. In addition, our model could be used to identify the optimal time for combination treatment, especially given the experimental observations in [12] and [13] suggest that combination therapies in which radiotherapy or chemotherapy are administered during the normalization window achieve better outcomes than when administered before or after the window.

Figure legends

Figure 1

Individual tumour size data for (a) control tumours (n=10), (b) bevacizumab treated animals (n=10), (c) vanucizumab treated animals (n=10), (d) mean tumour volume of individual groups. Treatment times are shown by vertical arrows in plots (b) - (d).

Figure 2

Typical model simulations with arbitrary parameters that are order of magnitude estimates. Curves on the left ((a), (c), (e)) represent the tumour volume $T(t)$ and corresponding curves on the right ((b), (d), (f)) represent the vessel dependent carrying capacity $V(t)$. (a)-(b) Control simulations (equations (1)-(2)) with $\alpha_T = 0.1$ day^{-1}, $\alpha_V = 0.09$ day^{-1}, $K = 2$, $T_0 = 70\text{mm}^3$. (c)-(d) Treatment model simulations (equations (3)-(5)) with $N_{max} = 1$, δ_V values 0.04 day^{-1} $-$ 0.12 day^{-1} and all other parameters the same as (a)-(b). (e)-(f) Treatment model simulations (equations (3)-(5)) where $t_{norm1} = 52$ days, $t_{norm2} = 62$ days, N_{max} values $2 - 21$, $\delta_V = 0.1$ day^{-1} and all other parameters the same as (a)-(b).

Figure 3

Monotonic model results from simulations of equations (3)-(5) where $N_{max} = 1$. (a)-(f) Two typical individual fits selected at random for (a)-(b) control; (c)-(d) bevacizumab, and (e)-(f) vanucizumab groups using experimental data. Key: Blue '+' - experimental data, solid black lines - predicted tumour volume, T, using individual parameters estimated by Monolix. (g) Experimental (observed) results for tumour volume plotted against predicted results for tumour volume for individuals and coloured by group. (h) Conditional weighted residuals (CWRES) plots for the transient dynamics model. The mean CWRES for each experimental group is shown by a solid line of the corresponding colour. Key: blue circles - control, green stars - bevacizumab, red dots - vanucizumab. The plots show that the tumour volume is consistently under-estimated for the treatment groups at day 52 and over-estimated at days 59 and 63.

Figure 4

Transient dynamics model results from simulations of equations (3)-(5). (a)-(f) Typical individual fits selected at random for (a)-(b) control; (c)-(d) bevacizumab, and (e)-(f) vanucizumab groups using experimental data. Key: Blue '+' - experimental data, solid black lines - predicted tumour volume, T, red dashed lines - normalization windows for individuals. (g) Experimental (observed) results for tumour volume plotted against predicted results for tumour volume for individuals and coloured by group. (h) Conditional weighted residuals (CWRES) plots for the transient dynamics model. Key: blue circles - control, green stars - bevacizumab, red dots - vanucizumab

Figure 5

Visual predictive check (VPC) for the final model (equations (3)-(5)) split by group. Parameters of the final model are listed in Table 1. Green, solid lines show the 10%, 50% and 90% quantiles of the observed data and the shaded regions represent the 90% prediction intervals on the theoretical 10% (blue region, lower), 50% (red region, middle) and 90% (blue region, upper) quantiles. Outliers are highlighted by red circles.

Figure 6

Model simulations for anti-angiogenic monotherapy, and three alternative theoretical treatment regimens to combine anti-angiogenic therapy and chemotherapy, equations (7)-(9). Key: Black lines - tumour volume (T), magenta lines - vessel dependent carrying capacity (V), dashed blue lines - intratumoural concentration of the cytotoxic drug, C. Blue shaded regions represent the normalization window and yellow shaded regions represent the delivery period of chemotherapy. (a) No chemotherapy, (b) chemotherapy administered before the transient window of enhanced tumour growth, (c) chemotherapy administered during the transient window of enhanced tumour growth, (d) chemotherapy administered for a three week period from day 38 to day 59. Anti-angiogenic therapy is administered from day 38 as in the preclinical study.

Acknowledgements

L.G.H acknowledges the financial support of the University of Oxford, the Systems Approaches to Biomedical Sciences Centre for Doctoral Training (SABS-CDT), the UK Engineering and Physical Sciences Research Council (EPSRC grant number EP/G037280/1) and F. Hoffman La-Roche Ltd.

Conflict of interest statement

AP, JW, CB and H-JM are employees and shareholders of Hoffman-La Roche.

Study Highlights

1. **What is the current knowledge on the topic?**

   Anti-angiogenic therapies alter the density and architecture of the tumour blood vessel network, and may stimulate a transient window of vessel normalization shortly after anti-angiogenic treatment commences. The efficacy of chemotherapy and radiotherapy may be enhanced during the transient window due to increased perfusion and decreased vascular permeability.

2. **What question did this study address?**

   Can tumour size data alone be used to infer the transient window of vessel normalization, in which the efficacy of chemotherapy and radiotherapy may be enhanced?

3. **What this study adds to our knowledge?**

   A transient window of enhanced tumour growth occurs during treatment with bevacizumab or vanucizumab for KPL-4 tumour bearing mice. The window is identified with precision using mixed effects techniques.

4. **How this might change drug discovery, development, and/or therapeutics?**

   Identification of the transient window of enhanced tumour growth could reduce the need to measure normalization parameters, and could reduce the chance of obtaining inconsistent efficacy measurements when comparing treatments to be administered alongside anti-angiogenic treatment.

References

[1] Sherwood LM, Parris EE & Folkman J. Tumor Angiogenesis: Therapeutic Implications. *New England Journal of Medicine* **285**(21), 1182–1186 (1971).

[2] Jain RK. Normalizing tumor vasculature with anti-angiogenic therapy: a new paradigm for combination therapy. *Nature Medicine* **7**(9), 987–989 (2001).

[3] Jain RK. Normalization of tumor vasculature: an emerging concept in antiangiogenic therapy. *Science* **307**(5706), 58–62 (2005).

[4] Myers AL, Williams RF, Ng CY, Hartwich JE & Davidoff AM. Bevacizumab-induced tumor vessel remodeling in rhabdomyosarcoma xenografts increases the effectiveness of adjuvant ionizing radiation. *Journal of pediatric surgery* **45**(6), 1080–5 (2010).

[5] Dickson PV *et al.* Bevacizumab-induced transient remodeling of the vasculature in neuroblastoma xenografts results in improved delivery and efficacy of systemically administered C chemotherapy. *Clinical Cancer Research* **13**(13), 3942–3950 (2007).

[6] Thomas M *et al.* A novel angiopoietin-2 selective fully human antibody with potent anti-tumoral and anti-angiogenic efficacy and superior side effect profile compared to Pan-Angiopoietin-1/-2 inhibitors. *PloS One* **8**(2), e54923 (2013).

[7] Kienast Y *et al.* Ang-2-VEGF-A CrossMab, a novel bispecific human IgG1 antibody blocking VEGF-A and Ang-2 functions simultaneously, mediates potent antitumor, antiangiogenic, and antimetastatic efficacy. *Clinical Cancer Research* **19**(24), 6730–6740 (2013).

[8] von Baumgarten L *et al.* Bevacizumab has differential and dose-dependent effects on glioma blood vessels and tumor cells. *Clinical cancer research : an official journal of the American Association for Cancer Research* **17**(19), 6192–205 (2011).

[9] Zhang Q, Bindokas V, Shen J, Fan H, Hoffman RM & Xing HR. Time-course imaging of therapeutic functional tumor vascular normalization by antiangiogenic agents. *Molecular cancer therapeutics* **10**(7), 1173–84 (2011).

[10] Carmeliet P & Jain RK. Principles and mechanisms of vessel normalization for cancer and other angiogenic diseases. *Nature Reviews Drug Discovery* **10**(6), 417–427 (2011).

[11] Goel S, Duda D, Xu L & Munn L. Normalization of the vasculature for treatment of cancer and other diseases. *Physiol Rev* **91**(3), 1071–1121 (2011).

[12] Winkler F *et al.* Kinetics of vascular normalization by VEGFR2 blockade governs brain tumor response to radiation: role of oxygenation, angiopoietin-1, and matrix metalloproteinases. *Cancer cell* **6**(6), 553–63 (2004).

[13] Dings RPM *et al.* Scheduling of radiation with angiogenesis inhibitors anginex and Avastin improves therapeutic outcome via vessel normalization. *Clinical cancer research : an official journal of the American Association for Cancer Research* **13**(11), 3395–402 (2007).

[14] Batchelor TT *et al.* AZD2171, a pan-VEGF receptor tyrosine kinase inhibitor, normalizes tumor vasculature and alleviates edema in glioblastoma patients. *Cancer cell* **11**(1), 83–95 (2007).

[15] Willett CG *et al.* Surrogate markers for antiangiogenic therapy and dose-limiting toxicities for bevacizumab with radiation and chemotherapy: continued experience of a phase I trial in rectal cancer patients. *Journal of clinical oncology : official journal of the American Society of Clinical Oncology* **23**(31), 8136–9 (2005).

[16] Dobosz M, Ntziachristos V, Scheuer W & Strobel S. Multispectral fluorescence ultramicroscopy : three- dimensional visualization and automatic quantification of tumor morphology , drug penetration , and antiangiogenic treatment response. *Neoplasia* **16**(1), 1–13, IN1–IN7 (2014).

[17] Zheng X, Young Koh G & Jackson T. A continuous model of angiogenesis: Initiation, extension, and maturation of new blood vessels modulated by vascular endothelial growth factor, angiopoietins, platelet-derived growth factor-B, and pericytes. *Discrete and Continuous Dynamical Systems - Series B* **18**(4), 1109–1154 (2013).

[18] Plank MJ, Sleeman BD & Jones PF. A mathematical model of tumour angiogenesis, regulated by vascular endothelial growth factor and the angiopoietins. *Journal of Theoretical Biology* **229**(4), 435–54 (2004).

[19] Lignet F *et al.* Theoretical investigation of the efficacy of antiangiogenic drugs combined to chemotherapy in xenografted mice. *Journal of Theoretical Biology* **320**, 86–99 (2013).

[20] Billy F *et al.* A pharmacologically based multiscale mathematical model of angiogenesis and its use in investigating the efficacy of a new cancer treatment strategy. *Journal of Theoretical Biology* **260**(4), 545–562 (2009).

[21] Perfahl H *et al.* Multiscale modelling of vascular tumour growth in 3D: the roles of domain size and boundary conditions. *PloS One* **6**(4), e14790–e14790 (2011).

[22] Scianna M, Bell CG & Preziosi L. A review of mathematical models for the formation of vascular networks. *Journal of Theoretical Biology* **333**, 174–209 (2013).

[23] Kuhn E & Lavielle M. Coupling a stochastic approximation version of EM with an MCMC procedure. *ESAIM: Probability and Statistics* **8**, 115–131 (2004).

[24] Simeoni M *et al.* Predictive Pharmacokinetic-Pharmacodynamic Modeling of Tumor Growth Kinetics in Xenograft Models after Administration of Anticancer Agents. *Cancer Research* **64**(3), 1094–1101 (2004).

[25] Ribba B *et al.* A tumor growth inhibition model for low-grade glioma treated with chemotherapy or radiotherapy. *Clinical cancer research : an official journal of the American Association for Cancer Research* **18**(18), 5071–80 (2012).

[26] Ribba B *et al.* A review of mixed-effects models of tumor growth and effects of anticancer drug treatment used in population analysis. *CPT: Pharmacometrics & Systems Pharmacology* **3**(5), 1–10 (2014).

[27] Wilson S *et al.* Modeling and predicting optimal treatment scheduling between the antiangiogenic drug sunitinib and irinotecan in preclinical settings. *CPT: Pharmacometrics & Systems Pharmacology* **4**(12), 720–727 (2015).

[28] Frances N, Claret L, Bruno R & Iliadis A. Tumor growth modeling from clinical trials reveals synergistic anticancer effect of the capecitabine and docetaxel combination in metastatic breast cancer. *Cancer Chemotherapy and Pharmacology* **68**(6), 1413–1419 (2011).

[29] Rocchetti M *et al.* Predictive pharmacokinetic-pharmacodynamic modeling of tumor growth after administration of an anti-angiogenic agent, bevacizumab, as single-agent and combination therapy in tumor xenografts. *Cancer chemotherapy and pharmacology* **71**(5), 1147–57 (2013).

[30] Hahnfeldt P, Panigrahy D, Folkman J & Hlatky L. Tumor development under angiogenic signaling: a dynamical theory of tumor growth, treatment response, and postvascular dormancy. *Cancer research* **59**(19), 4770–5 (1999).

[31] Ouerdani A, Struemper H, Suttle A, Ouellet D & Ribba B. Preclinical Modeling of Tumor Growth and Angiogenesis Inhibition to Describe Pazopanib Clinical Effects in Renal Cell Carcinoma. *CPT: Pharmacometrics & Systems Pharmacology* **4**(11), 660–668 (2015).

[32] Limpert E, Stahel WA & Abbt M. Log-normal Distributions across the Sciences: Keys and Clues. *BioScience* **51**(5), 341–352 (2001).

[33] Jain RK & Forbes NS. Can engineered bacteria help control cancer? *Proceedings of the National Academy of Sciences of the United States of America* **98**(26), 14748–50 (2001).

[34] Lin YS *et al.* Preclinical Pharmacokinetics, Interspecies Scaling, and Tissue Distribution of a Humanized Monoclonal Antibody against Vascular Endothelial Growth Factor. *J. Pharmacol. Exp. Ther.* **288**(1), 371–378 (1999).

[35] Kuhn E & Lavielle M. Maximum likelihood estimation in nonlinear mixed effects models. *Computational Statistics & Data Analysis* **49**(4), 1020–1038 (2005).

[36] Tong RT, Boucher Y, Kozin SV, Winkler F, Hicklin DJ & Jain RK. Vascular normalization by vascular endothelial growth factor receptor 2 blockade induces a pressure gradient across the vasculature and improves drug penetration in tumors. *Cancer research* **64**(11), 3731–6 (2004).
