# Supplementary material for: Modeling Longitudinal Preclinical Tumor Size Data to Identify Transient Dynamics in Tumor Response to Antiangiogenic Drugs
Source: CPT Pharmacometrics Syst Pharmacol. 2016 Nov 14;5(11):636–45. doi: 10.1002/psp4.12142 (PMC5192995; doi:10.1002/psp4.12142)
Supplement: Supplementary file 2 — Supporting Information [file PSP4-5-636-s002.pdf]

Supplementary material 1

Synthetic data

We generated synthetic data for T and V by solving equations (1)-(2) for the control model and equations (3)-(5) for the treatment model, using parameter values from known distributions, with added noise to represent the experimental measurement error. The magnitude of the noise was proportional to the tumour size at that time, i.e. we used a proportional error model. As before, the datasets contain values of T and V at times $t = 38, 42, 45, 49, 52, 56, 59, 63, 67, 71$ days. The true parameter distributions that were sampled to generate the data are listed in Table 1.

Parameter	Distribution	Mode (pop)	Standard Deviation (ω)
T_0 (not estimated)	uniform(50,90)	–	–
α_T	lognormal	0.1	0.3
α_V	lognormal	0.09	0.3
K	lognormal	2	0.845
t_{norm1}	none	52	0
t_{norm2}	none	62	0
N_{max}	lognormal	10	0.3
δ_V	lognormal	0.1	0.3
b	none	0.2	–
ϵ	normal	0	b

Table 1: The distributions of parameters used to generate the synthetic data. A proportional error model was used such that $y = f + f\ b\ \epsilon$ and $\epsilon = \mathcal{N}(0, 1)$.

The synthetic datasets were of size $N = 10$ per group or $N = 100$ per group, and we considered 3 values of the proportional error parameter: $b = 0.0$, 0.1 and 0.2. We also attempted to fit the model parameters using data on tumour volume alone, and using simultaneous data on T and V. In total, 12 datasets were generated.

Figure 1 shows the results of the simulations for each dataset. From figure 1, it is clear that the r.s.e. values are largest at all error levels for the $N_{max}(pop)$ and $N_{max}(\omega)$ parameters: from equation (3), when $N \times V \gg T$, $\frac{\mathrm{d}T}{\mathrm{d}t} \approx \alpha_T T$ and for simulations where N is large, T behaves in a similar manner since α_T is a constant. As a result, the likelihood is relatively insensitive to N_{max}. In most cases, r.s.e. values decrease as more data is used for the simulations. In almost all cases, fitting with T and V data results in more accurate parameter estimates than fitting T data only. From our simulations, when $b = 0.1$, the parameter estimates are more accurate than when $b = 0.2$, but there is little difference between the accuracy of the results for $b = 0.1$ and $b = 0.0$. This suggests that parameter estimates can be improved by reducing experimental error.

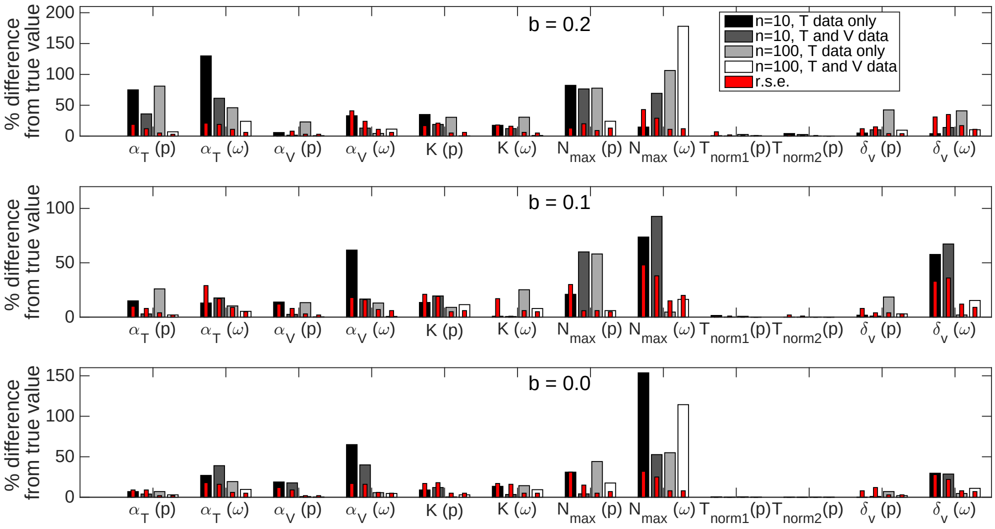

Figure 1: Bar charts showing how the percentage difference between parameter estimates and the true parameter values and the r.s.e. change as different datasets are used. The r.s.e. of parameter estimates decreases as the amount of data increases, and in general, using T and V gives more reliable estimates than using T only.
