# Supplementary material for: Modeling Longitudinal Preclinical Tumor Size Data to Identify Transient Dynamics in Tumor Response to Antiangiogenic Drugs
Source: CPT Pharmacometrics Syst Pharmacol. 2016 Nov 14;5(11):636–45. doi: 10.1002/psp4.12142 (PMC5192995; doi:10.1002/psp4.12142)
Supplement: Supplementary file 3 — Supporting Information [file PSP4-5-636-s003.pdf]

Supplementary material 1: Results for the population parameters, inter-individual variability and r.s.e. values for simulations using combinations of fixed and free parameters. The first 4 rows indicate the parameters (of δ_V, N_max and T_norm1,2) that have been defined as treatment specific in each model. Population values of parameters are indicated by _pop, standard deviations of population distributions for each parameter are indicated by _omega and residual standard error values are indicated by _rse. Where a parameter applies only to one treatment group, this is indicated by _bev or _van. Model selection was performed by comparing the BIC values (where appropriate), diagnostic plots and the r.s.e. of parameter estimates. The selected model is highlighted in yellow.

Parameter	Model 1	Model 2	Model 3	Model 4	Model 5	Model 6	Model 7	Model 8
delta_V treatment specific?	Y	Y	N	N	Y	Y	Y	Y
N_max treatment specific?	Y	N	Y	N	N	N	N	N
Tnorm treatment specific?	N	N	N	N	Y	Y	Y	Y
Tnorm IIV fixed to 0?	Y	Y	Y	Y	Y	N	N	N
α_T_pop	0.0834	0.108	0.0979	0.0808	0.101	0.109	0.111	0.0968
α_T_pop_rse	8	10	10	8	9	12	8	7
α_T_omega	0.264	0.299	0.297	0.23	0.359	0.422	0.273	0.237
α_T_omega_rse	23	32	29	29	21	25	25	26
α_V_pop	0.133	0.11	0.106	0.179	0.107	0.119	0.0734	0.0987
α_V_pop_rse	9	7	8	8	3	7	6	7
α_V_omega	0.178	0.142	0.183	0.201	0.0661	0.177	0.204	0.202
α_V_omega_rse	40	44	36	31	36	34	27	31
K_pop	1.39	1.2	1.31	1.48	1.23	1.14	1.18	1.23
K_pop_rse	18	14	16	21	14	15	13	14
K_omega	0.9	0.723	0.795	1.04	0.749	0.711	0.666	0.74
K_omega_rse	15	15	15	15	13	15	14	14
N_max_pop	na	0.11	na	354	2.72	6.7	6.71	15.7
N_max_pop_rse	na	8	na	68	11	27	24	20
N_max_omega	na	0.231	na	1.57	0.459	0.55	0.935	0.63
N_max_omega_rse	na	66	na	31	18	41	20	26
N_max_bev_pop	626	na	13.2	na	na	na	na	na
N_max_bev_pop_rse	64	na	26	na	na	na	na	na
N_max_bev_omega	0.824	na	0.525	na	na	na	na	na
N_max_bev_omega_rse	59	na	38	na	na	na	na	na
N_max_van_pop	664	na	199	na	na	na	na	na
N_max_van_pop_rse	84	na	125	na	na	na	na	na
N_max_van_omega	1.63	na	2.77	na	na	na	na	na
N_max_van_omega_rse	38	na	33	na	na	na	na	na
Tnorm1_pop	53	53	53.6	52.6	na	na	na	na
Tnorm1_pop_rse	2	1	1	1	na	na	na	na
Tnorm1_omega	FIX 0	FIX 0	FIX 0	FIX 0	na	na	na	na
Tnorm1_omega_rse	na	na	na	na	na	na	na	na
Tnorm2_pop	61.4	61.8	61.4	61.5	na	na	na	na
Tnorm2_pop_rse	1	1	2	1	na	na	na	na
Tnorm2_omega	FIX 0	FIX 0	FIX 0	FIX 0	na	na	na	na
Tnorm2_omega_rse	na	na	na	na	na	na	na	na
Tnorm1_bev_pop	na	na	na	na	51.7	53.3	53.3	52.6
Tnorm1_bev_rse	na	na	na	na	4	4	3	6
Tnorm1_bev_omega	na	na	na	na	FIX 0	FIX 0.1	0.0352	FIX 0.1
Tnorm1_bev_omega_rse	na	na	na	na	na	na	115	na
Tnorm2_bev_pop	na	na	na	na	62.3	59.2	61.9	59.6
Tnorm2_bev_rse	na	na	na	na	2	3	2	4
Tnorm2_bev_omega	na	na	na	na	FIX 0	FIX 0.1	0.0289	FIX 0.1
Tnorm2_bev_omega_rse	na	na	na	na	na	na	63	na
Tnorm1_van_pop	na	na	na	na	54.5	na	54.7	53.8
Tnorm1_van_rse	na	na	na	na	204	na	4	nan
Tnorm1_van_omega	na	na	na	na	FIX 0	na	0.0753	FIX 0.1
Tnorm1_van_omega_rse	na	na	na	na	na	na	41	na
Tnorm2_van_pop	na	na	na	na	60	na	56.4	49.4
Tnorm2_van_rse	na	na	na	na	34	na	9	nan
Tnorm2_van_omega	na	na	na	na	FIX 0	na	0.0335	FIX 0.1
Tnorm2_van_omega_rse	na	na	na	na	na	na	721	na
δ_V_pop	na	na	0.114	0.213	na	na	na	na
δ_V_pop_rse	na	na	9	7	na	na	na	na
δ_V_omega	na	na	0.198	0.115	na	na	na	na
δ_V_omega_rse	na	na	34	59	na	na	na	na
δ_V_bev_pop	0.144	0.11	na	na	0.114	0.113	0.0719	0.0854
δ_V_bev_pop_rse	11	8	na	na	8	11	8	15
δ_V_bev_omega	0.0693	0.128	na	na	0.191	0.208	0.118	0.33
δ_V_bev_omega_rse	253	50	na	na	35	47	66	39
δ_V_van_pop	0.159	0.123	na	na	0.106	0.115	0.0652	0.0909
δ_V_van_pop_rse	13	11	na	na	12	12	17	17
δ_V_van_omega	0.231	0.248	na	na	0.301	0.293	0.409	0.436
δ_V_van_omega_rse	46	33	na	na	31	33	33	30
