# Supplementary material for: Modeling Longitudinal Preclinical Tumor Size Data to Identify Transient Dynamics in Tumor Response to Antiangiogenic Drugs
Source: CPT Pharmacometrics Syst Pharmacol. 2016 Nov 14;5(11):636–45. doi: 10.1002/psp4.12142 (PMC5192995; doi:10.1002/psp4.12142)
Supplement: Supplementary file 5 — Supporting Information [file PSP4-5-636-s005.pdf]

# Supplementary Material 4

## Comparison with histology data

We wish to validate our model by comparing our estimate of the vessel dependent carrying capacity, $V$, at the end of the model simulations (day 71) with histological data (tumours harvested at day 71) for vessel density. Vessel number and size were automatically quantified (Definiens) on entire slides (5 tumours per group) and microvessel density was calculated as vessels per $mm^2$ viable tissue. Since our simulation results for the vessel dependent carrying capacity are measured as a volume ($mm^3$), we can only qualitatively compare with the density results from histology.

We compared the vessel density from histology with the effective vessel density from our model, calculated as $V/T$. For the histology and simulated data, we scaled our results by dividing by the vessel density for the control group. The results are shown in Figure 1.

The results for the simulated bevacizumab and vanucizumab vessel dependent carrying capacities are in good qualitative agreement with the histology results, since according to our model and the histology data, vanucizumab treatment leads to a lower vessel density than bevacizumab treatment. In our simulations, $V$ corresponds to the vascular-dependent carrying capacity of the tumour, and theoretically, this includes the vasculature within the tumour and surrounding it. For the histology results, only vessels inside viable tumour regions were quantified: this could explain the discrepancy between the histology and simulation results for the experimental groups. Overall there is good qualitative agreement between simulations of anti-angiogenic treatment and histology results.

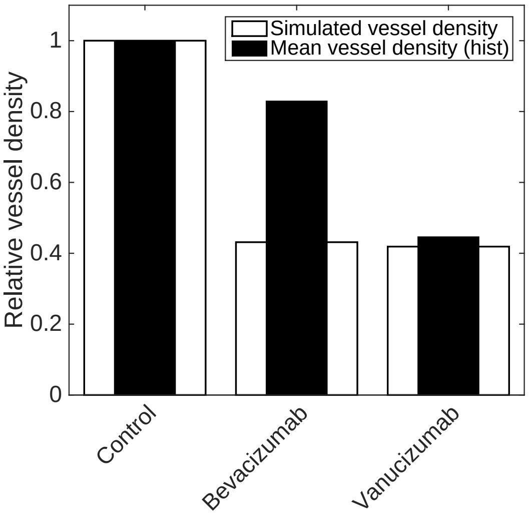


Figure 1: A bar chart to compare the simulated vessel density, $V/T$, at day 71 to the vessel density at day 71 from histology data, which was measured as vessels per $mm^2$. Each experimental group has been normalized by dividing by the vessel density for the control group.
