# Supplementary material for: Modeling Longitudinal Preclinical Tumor Size Data to Identify Transient Dynamics in Tumor Response to Antiangiogenic Drugs
Source: CPT Pharmacometrics Syst Pharmacol. 2016 Nov 14;5(11):636–45. doi: 10.1002/psp4.12142 (PMC5192995; doi:10.1002/psp4.12142)
Supplement: Supplementary file 6 — Supporting Information [file PSP4-5-636-s006.pdf]

Supplementary Material 5

Sensitivity of the chemotherapy simulations to the parameter λ

The conclusions made in the main text regarding the hypothetical concomitant therapy schedules depend heavily on several assumptions. The parameters for these simulations were taken directly from the study in [28]. The parameter λ represents the emergence of resistance to chemotherapy and therefore plays a key role in the conclusions drawn from our study. Here we perform simulations of chemotherapy regimens similar to those in Section 3.4 in the main text for the cases that λ is 20% smaller or 20% larger than the default value, $\lambda_{default}$. In the main text we conclude from Figure 6 that the tumour size at day 71 is smaller when chemotherapy is administered only within the normalization window (days 54-61) compared to when it is administered from days 38-59. Figures 1 and 2 show that when $\lambda = 0.8\lambda_{default}$ or $\lambda = 1.2\lambda_{default}$, the same conclusion holds.

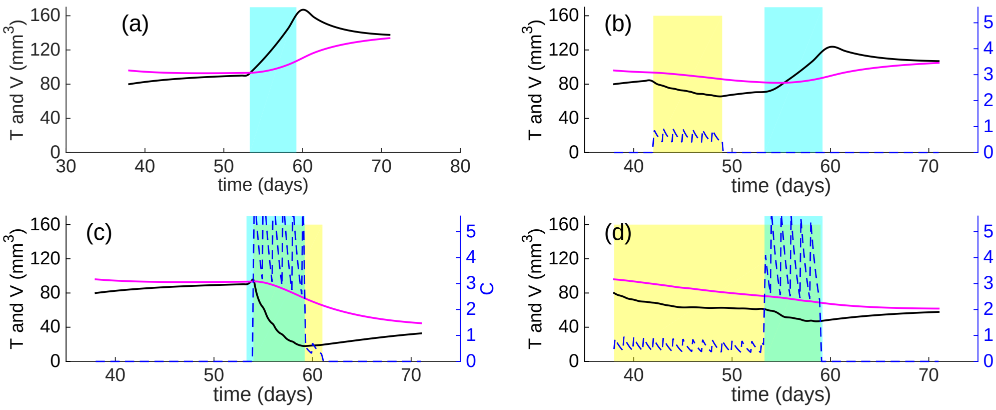

Figure 1: Model simulations for anti-angiogenic monotherapy, and three alternative theoretical treatment regimens to combine anti-angiogenic therapy and chemotherapy, equations (7)-(9). Key: Black lines - tumour volume (T), magenta lines - vessel dependent carrying capacity (V), dashed blue lines - intratumoural concentration of the cytotoxic drug, C. Blue shaded regions represent the normalization window and yellow shaded regions represent the delivery period of chemotherapy. (a) No chemotherapy, (b) chemotherapy administered before the transient window of enhanced tumour growth, (c) chemotherapy administered during the transient window of enhanced tumour growth, (d) chemotherapy administered for a three week period from day 38 to day 59. Anti-angiogenic therapy is administered from day 38 as in the preclinical study. For this simulation, $\lambda = 0.8\lambda_{default}$.

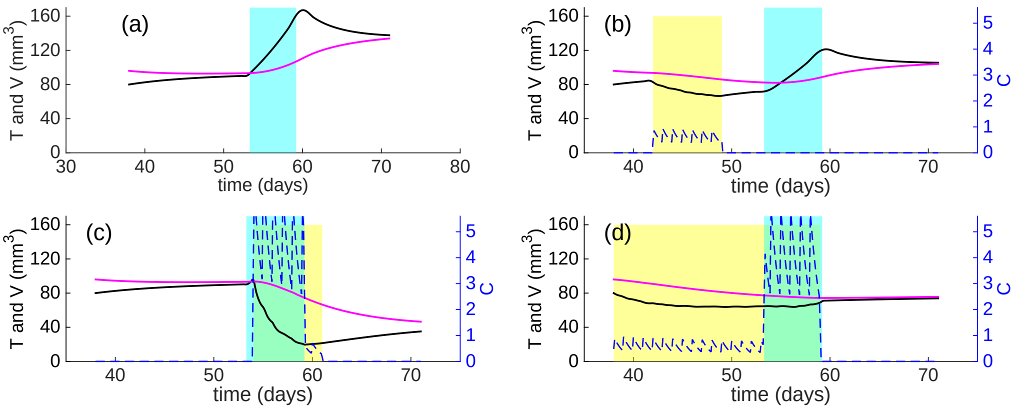

Figure 2: Model simulations for anti-angiogenic monotherapy, and three alternative theoretical treatment regimens to combine anti-angiogenic therapy and chemotherapy, equations (7)-(9). Key: Black lines - tumour volume (T), magenta lines - vessel dependent carrying capacity (V), dashed blue lines - intratumoural concentration of the cytotoxic drug, C. Blue shaded regions represent the normalization window and yellow shaded regions represent the delivery period of chemotherapy. (a) No chemotherapy, (b) chemotherapy administered before the transient window of enhanced tumour growth, (c) chemotherapy administered during the transient window of enhanced tumour growth, (d) chemotherapy administered for a three week period from day 38 to day 59. Anti-angiogenic therapy is administered from day 38 as in the preclinical study. For this simulation, $\lambda = 1.2\lambda_{default}$.
